# Patient involvement in guidelines is poor five years after institute of medicine standards: review of guideline methodologies

**DOI:** 10.1186/s40900-017-0070-2

**Published:** 2017-10-02

**Authors:** Melissa J. Armstrong, Joshua A. Bloom

**Affiliations:** 1 Department of Neurology, University of Florida College of Medicine, Gainesville, FL USA; 2 University of Florida College of Medicine, Gainesville, FL 32610 USA

**Keywords:** Guidelines, Patient participation, Patient engagement, Patient-centered care

## Abstract

**Plain English summary:**

The 2011 standards for trustworthy development of healthcare guidelines published by the United States-based Institute of Medicine recommend that guideline developers involve patients and public representatives in the development process. The standards recommend that (1) patients and the public be actively involved as members on guideline development panels and (2) guideline developers seek patient and public input during review of the draft guideline. In this study, researchers reviewed the patient and public involvement strategies of guideline developers in the United States by looking at websites and guideline development practices. Of 101 organizations reviewed, only 8% require patient and public involvement on guideline development groups; 15% sometimes require it or describe it as optional. Only 24% of guideline developers always post draft guidelines for public comment. Thirteen percent of guideline developers ask patients or patient organizations to review draft guidelines at least some of the time. Only 20% of guideline developers create patient-targeted guideline products (e.g. patient summaries of guidelines). These low numbers show that there is a substantial gap between standards for patient and public involvement in guideline development and what is actually happening. This is a missed opportunity, as patient and public contributions to guideline development include assessing guideline priorities, introducing new topics, identifying important populations and outcomes, suggesting whether findings are meaningful, prompting holistic approaches to care, assessing how recommendations interact with patient values, and writing plain-language guideline versions. Guideline developers must commit to prioritizing patient and public involvement as one part of trustworthy guideline development.

**Abstract:**

**Background:**

The United States-based Institute of Medicine 2011 standards for trustworthy clinical practice guideline development recommended patient and public involvement in guideline development via participation by patients and public representatives on guideline development groups and via external review and public comment strategies. Guideline developer compliance with these standards has not been assessed. This study aimed to identify the frequency with which United States guideline developers are employing participation, consultation, and communication patient and public involvement strategies.

**Methods:**

Two reviewers independently extracted current patient and public involvement strategies of independent guideline developers, either (1) an organizational member of the Guidelines-International-Network North America and/or (2) having ≥2 guidelines in the National Guideline Clearinghouse between March 2011 and November 2015. Publicly available information was extracted from guideline developers’ websites, methodology manuals, and guidelines between November 2015 and December 2016. Disagreements were resolved by discussion.

**Results:**

Of 101 organizations meeting inclusion criteria, only 8% require patient/public involvement on guideline development groups; 15% sometimes require it or describe it as optional. Only 24% always utilize public comment on draft guidelines; 13% engage patients/public in external review at least some of the time. Twenty percent of developers create patient-targeted guideline products.

**Conclusions:**

There remains a substantial gap between patient/public involvement standards for guideline development and practice in the United States, even 5 years after publication of Institute of Medicine standards. This is a missed opportunity, as patient and public contributions to guideline development include assessing guideline priorities, introducing new topics, identifying key populations and outcomes, informing whether findings are meaningful, prompting holistic approaches to care, assessing how recommendations interact with patient values, and writing plain-language guideline versions. Guideline developers must commit to prioritizing patient and public involvement as one element of trustworthy guideline development.

**Electronic supplementary material:**

The online version of this article (doi:10.1186/s40900-017-0070-2) contains supplementary material, which is available to authorized users.

## Background

In 2011, the United States-based Institute of Medicine (IOM) released “Clinical Practice Guidelines We Can Trust,” outlining standards for guideline development [1]. This report identifies eight standards of trustworthy clinical practice guidelines including transparency, managing conflicts of interest, use of systematic reviews, processes for recommendation development, and updating mechanisms. Patient and public involvement (PPI) is highlighted in multiple standards. Standard 3 emphasizes that guideline development groups should include populations impacted by the guideline and states that “patient and public involvement should be facilitated by including (at least at the time of clinical question formulation and draft CPG [clinical practice guideline] review) a current or former patient and a patient advocate or patient/consumer organization representative” on the guideline development group [1]. Standard 7.1 requires that external reviewers should include patients and representatives of the public; Standard 7.4 requires that a guideline draft be made available to the general public for comment after “reasonable notice of impending publication” and before final publication [1].

This emphasis on increased PPI is consistent with other organizations advising on guideline development, though recommended and required mechanisms vary. The IOM and Guidelines-International-Network (G-I-N) recommend that patients and/or consumers actively participate on guideline development groups [1, 2], whereas the Appraisal of Guidelines for Research and Evaluation II (AGREE II) instrument for evaluating guidelines simply requires that guideline developers seek the views of the target population in addition to including representative professionals on guideline development groups [3].

External review as a mechanism for improving guideline quality is recommended by the IOM, AGREE II, and G-I-N, with AGREE-II focusing on expert review [3], G-I-N describing external stakeholders which “may” include members of the public [2], and the IOM requiring patient and public engagement in both external review and public comment [1].

These mechanisms reflect two of three described strategies for PPI in guidelines – (1) participation, where patients or consumers join *with* the guideline development group as members, (2) consultation, which includes various strategies (including public comment, focus groups, surveys) to obtain views *from* large numbers of individuals, and (3) communication, where information flows from the developer *to* patients and the public (usually as part of dissemination and implementation strategies) to enhance guideline uptake and implementation [4].

While there is international consensus on the importance of PPI in guideline development, there is little research investigating whether this is actually occurring. The only known report addressing this is a 2008 survey of international guideline developers, where 39% of 31 guideline developers reported involving consumers (patients or the general public) through participation on a guideline development group, 29% surveyed consumers for views and preferences, and 45% involved consumers in reviewing draft guidelines. Only 29% of guideline developers always involved consumers and 39% reported involving consumers “only if necessary” [5]. No studies investigate more recent trends in PPI in guideline development or whether U.S. guideline developers are including PPI as recommended by the 2011 IOM report.

It is important to investigate current PPI practices to know whether there is a need for additional strategies to improve PPI in guideline development. In this study, we aimed to identify the frequency with which U.S. guideline developers follow IOM standards for guideline development, now 5 years after the standards’ publication. We considered both the IOM standards and G-I-N PUBLIC toolkit as framing mechanisms, looking at active PPI in guideline development groups (Standard 3, participation), external review and public comment (Standard 7, consultation), and the production of patient- and public-targeted guideline products (communication).

## Methods

### Participants

No Institutional Review Board approval was needed as only publicly available organizational website data was utilized. Guideline developers were identified from two sources: G-I-N North America (organizational members as of 10/16/2015) and the National Guideline Clearinghouse (NGC). The NGC permits submissions by international guideline developers but contributors are primarily based in the United States. Inclusion criteria were: (1) an organizational member of G-I-N North America (queried 10/16/2015) or a developer with ≥2 guidelines in the NGC between March 1, 2011 and November 25, 2015, inclusive, and (2) an independent guideline developer. Because the intent was to identify practices of active guideline developers (so as not to artificially lower estimated PPI frequency by including organizations who rarely produce guidelines), organizations with only one guideline in the NGC since 2011 were excluded. G-I-N North America organizational members that help societies develop guidelines but do not have internal methodologies of their own were excluded, as were organizations in the NGC that only adapt guidelines or collaborate on others’ guidelines with no independent guideline process.

### Data extraction

A data extraction form [see Additional file 1] was created and pilot tested by three extractors for consistency before proceeding with full extraction. Two reviewers independently extracted the information for each guideline developer between November 2015 and December 2016. When reviewers completed spreadsheet cells differently, websites were re-reviewed and discussed to achieve consensus.

Information was preferentially extracted from the most recent publicly available website content and links to guideline development manuals. If the required information was not identified from those two sources and the website included a link to published guidelines, extractors were instructed to use the most recently linked guideline for evidence of methodology. Because the most recently published guideline was not easily identifiable for all developers, in cases where methodology was assessed by reviewing a published guideline, each reviewer noted the guideline that he or she used for this purpose. Knowing the guideline used to assess methodology assisted in reconciling differences in data extraction. No effort was made to contact guideline developers to obtain additional information and no for-purchase publications were reviewed. This approach was chosen a priori because IOM Standard 1 states that the guideline development process should be stated explicitly and publicly available [1]. Information available on the NGC website alone was not considered sufficient.

Extracted information [see Additional file 2] included details regarding PPI on guideline development groups, posting of protocols for public comment, distribution of draft guidelines for external review and/or public comment, and development of patient- and public-facing guideline products.

### Definitions

For this study, “guidelines” were defined as guideline-type publications that included recommendations based on an evidence review. This included products labeled guidelines, practice advisories, etc., but not consensus-based guidelines or systematic reviews without accompanying recommendations. “External review” was defined as a process where reviewers from outside the development process were specifically solicited to critically review the guideline prior to publication. Society board/committee review and journal editorial review were not considered sufficient for external review, a decision made a priori based on IOM guidance. “Public comment” was defined as posting of the guideline for comment by the general public prior to publication. Assessments of external review and public comment were based on these definitions and not developer terminology. For example, if a developer described “public comment” but access was via a member-only site, this was not considered “public comment” for the purposes of this study. Only posting of the documents prior to publication was considered sufficient as per the IOM standard.

Patient and public-facing materials that were guideline summaries or specifically mentioned the guideline were considered guideline-related. Many organizations had patient educational materials, sometimes overlapping with guideline topics, and these alone were not considered guideline-related.

### Analysis

The analysis was primarily descriptive in nature. When information about a particular question was not identified, it was assumed that it did not occur. Given the low identified frequency of PPI practices and the fact that an aim of G-I-N North America is to “improve the effectiveness, rigor and efficiency of guideline development” in the North American community [6], a post hoc analysis compared differences in PPI practices between developers who were and were not G-I-N North America members to inform potential trends in guideline development. In this analysis, the frequency of engagement practices of G-I-N North America organizational members (grouping all members of G-I-N North America together regardless of whether or not they contribute to the NGC) and contributors to the NGC alone were compared using risk differences (RDs) with 95% confidence intervals (CIs) calculated using Wilson’s method.

## Results

**Fig. 1:**
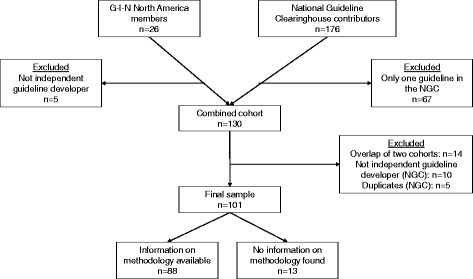
Flow Diagram of Selection of Independent Guideline Developers for Inclusion

In October 2015, there were 26 organizational members of G-I-N North America, five of which were excluded because they were not independent guideline developers. The NGC search for organizations publishing guidelines between March 1, 2011 and November 25, 2015 resulted in 176 organizations, 67 of which were excluded because they only had one guideline in the NGC (Fig. 1). Seven of the 21 organizations included as G-I-N North America members did not meet NGC criteria (four were not in the NGC list and three had only one guideline in the NGC) but were included given the G-I-N North America inclusion criterion. After removing duplicates and organizations that were not independent guideline developers, there were 101 guideline developers available for review (Fig. 1): 7 G-I-N North America members, 80 developers from the NGC, and 14 developers from both sources. All included developers appeared to be based in the United States, though some organizations had North American or international scope. There were 13 developers (12 from the NGC and 1 from G-I-N North America) where no information on their process could be identified because their methods and guidelines were behind a firewall, in-print only, or not provided.

Most guideline developers represented specialty or subspecialty organizations (Table 1). Guideline manuals (either formal manuals or sufficient website information to be considered equivalent to a manual) were available for 44 (44%) of developers.

**Table 1:** Characteristics of included guideline developers

	Total	G-I-N NA	G-I-N NA + NGC	NGC
Specialty society/ organization	83	6	12	65
University, hospital, medical system, or medical provider	9	1	1	7
Unaffiliated guideline developer	3	0	1	2
Government organization (national or state)	6	0	0	6
Total	101	7	14	80

*G-I-N NA* Guidelines International Network North America chapter, *NGC* National Guideline Clearinghouse

Only 8 (8%) guideline developers require PPI on guideline development groups and an additional 15 (15%) sometimes require it or describe it as optional. PPI on guideline development groups (defined as engaging patients at least sometimes) is significantly more common for developers participating in G-I-N North America than for contributors to the NGC alone (12/21 vs 11/80, RD 43%, 95% CI 21% to 63%).

Only 6 developers post guideline development protocols for public comment prior to project initiation at least sometimes (Table 2). Only one of the organizations, the United States Preventive Services Task Force (USPSTF), posts a protocol (“draft research plan”) that uses a template that is public-friendly (plain language, straight-forward questions for comment, methodologic information in tabular view, no excess background/technical information).

**Table 2:** Approaches to external review and public comment

	Total (%), *n* = 101	G-I-N NA alone (%), *n* = 7	G-I-N NA + NGC (%), *n* = 14	NGC alone (%), *n* = 80
Posts protocol for public comment	No: 95 (94%)	No: 7 (100%)	No: 12 (86%)	No: 76 (95%)
	Sometimes: 3 (3%)	Sometimes: 0 (0%)	Sometimes: 2 (14%)	Sometimes: 1 (1%)
	Yes: 3 (3%)	Yes: 0 (0%)	Yes: 0 (0%)	Yes: 3 (4%)
Obtains external review of draft guideline	No: 43%	No: 1 (14%)	No: 3 (21%)	No: 39 (49%)
	Sometimes: 6 (6%)	Sometimes: 1 (14%)	Sometimes: 1 (7%)	Sometimes: 4 (5%)
	Yes: 52 (51%)	Yes: 5 (71%)	Yes: 10 (71%)	Yes: 37 (46%)
Patients represented in external review of draft guideline	No or uncertain: 88 (77%)	No or uncertain: 6 (86%)	No: 12 (86%)	No: 70 (87.5%)
	Sometimes: 6 (6%)	Sometimes: 0 (0%)	Sometimes: 2 (14%)	Sometimes: 4 (5%)
	Yes: 7 (7%)	Yes: 1 (14%)	Yes: 0 (0%)	Yes: 6 (7.5%)
Posts draft guideline for public comment	No: 75 (74%)	No: 4 (57%)	No: 8 (57%)	No: 63 (79%)
	Sometimes: 2 (2%)	Sometimes: 0 (0%)	Sometimes: 1 (7%)	Sometimes: 1 (1%)
	Yes: 24 (24%)	Yes: 3 (43%)	Yes: 5 (36%)	Yes: 16 (20%)
Patients represented in external review OR public comment	No or uncertain: 68 (67%)	No or uncertain: 3 (43%)	No or uncertain: 7 (50%)	No or uncertain: 58 (72.5%)
	Yes (at least sometimes): 33 (33%)	Yes (at least sometimes): 4 (57%)	Yes (at least sometimes): 7 (50%)	Yes^a^ (at least sometimes): 22 (27.5%)

*G-I-N NA* Guidelines International Network North America chapter, *NGC* National Guideline Clearinghouse

^a^Includes 3 developers for which one response was “sometimes” and the other was “no”

Only half of U.S. guideline developers have an established process for obtaining external review and only 13% have evidence of patient or public engagement in external review at least some of the time (Table 2). Some developers describe guideline committee, society board, or journal peer-review, but these were considered insufficient for external review in this study. External review in general is more commonly performed by G-I-N North America members than contributors to the NGC alone (17/21 vs 41/80, RD 30%, 95% CI 6% to 45%), but there is no significant difference between groups when considering PPI in external review (3/21 vs 10/80, RD 2%, 95% CI -11% to 23%).

Public comment is routinely performed by 24% of U.S. guideline developers and sometimes performed by an additional 2% (Table 2). There is no difference in the number of guideline developers requiring public comment for all guidelines between G-I-N North America members and contributors to the NGC (8/21 vs 16/80, RD 18%, 95% CI -2% to 40%). G-I-N North America members have a slightly higher frequency of performing public comment at least sometimes (9/21 vs 17/80, RD 22%, 95% CI 0.6% to 44%), with confidence intervals including values of limited clinical importance. There is no evidence that any guideline developer posts patient-friendly versions of the draft guideline for comment.

While not IOM-compliant, if either PPI in external review or public comment is accepted as sufficient, then PPI via at least one of these consultation strategies is more commonly performed by G-I-N North America members than contributors to the NGC alone (11/21 vs 22/80, RD 25%, 95% CI 2% to 46%) (Table 2).

For organizations describing the length of external review, the time provided ranges from 2 days (where feedback is given at a professional meeting) to 4 months. Almost half of developers (6/13) describing the length of external review report a 4 week timeframe. For public comment, one developer describes using a public hearing for feedback. Fifteen other developers describe a period of weeks allotted for public comment, 8 of which use a 4 week time frame (range 14–60 days).

Only 20% of U.S. guideline developers prepare patient/public versions of guidelines or guideline summaries at least some of the time, with no difference between G-I-N North America members and NGC contributors alone (7/21 vs 13/80, RD 17%, 95% CI -2% to 39%). During the conduct of the study there was no identified evidence that any developer creating patient/public versions engages patients or the public in that process. Subsequently authors became aware that the American Academy of Otolaryngology-Head and Neck Surgery includes patient/public representatives as co-authors on plain language summaries, an approach not captured in their methodology manual.

## Discussion

Five years after publication of the IOM’s standards for trustworthy guidelines, there is still substantial room for improvement in PPI in guideline development in the U.S. PPI on guideline development groups is uncommon, required by only 8% of U.S. guideline developers. When considering consultation strategies, only 6 U.S. guideline developers post protocols for public comment and only the USPSTF does it in a way that is public-friendly. At the draft guideline stage, external review is performed routinely by only half of guideline developers and only 13% engage patients or the public at least some of the time. Only a quarter of developers post drafts for public comment. Communication PPI strategies are similarly uncommon, with only 20% of U.S. guideline developers preparing patient guidelines or summaries at least some of the time.

These numbers are discouragingly low, particularly since 5 years have passed since publication of the IOM report. They are also even lower than a 2008 survey of international guideline developers, where 39% of 31 guideline developers reported involving consumers (patients or the general public) through participation on a guideline development group, 29% surveyed consumers for views and preferences, and 45% involved consumers in reviewing draft guidelines. However, only 29% of guideline developers always involved consumers and 39% reported involving consumers “only if necessary” [5].

If guideline developers who join G-I-N North America are those most likely to be early-adopters and/or leaders in guideline methodology, then the fact that G-I-N North America members are more likely to have PPI in some categories may suggest that the guideline field is slowly moving in this direction. The frequency of PPI is low even for G-I-N North America members, however, regardless of PPI strategy.

The lack of PPI in guidelines is a missed opportunity. While stated rationales for PPI are largely ethical ones – emphasizing patients’ autonomy and experiential knowledge in the context of person-centered healthcare, consumer rights, and/or democratic rights of citizens and taxpayers [4] – increasing evidence suggests that patients and the public make meaningful contributions to guideline development. PPI at the step of question and protocol development and review can lead to the inclusion of new topics, identification of key special populations of interest and patient-relevant outcomes, elaboration of scope, and further development of the analytic framework [7–11]. The GRADE Evidence to Decision framework notes that PPI at the step of recommendation development can help identify whether the problem is a priority, inform whether effects are meaningful, weigh risks and benefits, analyze the impact of costs, and assess acceptability and feasibility [12], in line with IOM standards for developing recommendations. PPI can also prompt guideline developers to have a more holistic approach to recommendations, including covering topics such as support for families and caregivers, patient education, self-management, and non-pharmacologic options [11]. At the implementation and dissemination stage, patients and the public can help with development of plain-language guideline versions or summaries [7, 11, 13].

Known barriers likely contribute to the low frequency of PPI in guidelines. Identified barriers include limited resources [14], uncertainty of how to incorporate patient experiences into evidence-based guidelines [14], the commitment required (work, time) [13], recruitment difficulties [13], challenges in patients and the public understanding medical terminology and participating meaningfully in assessing research quality [2, 11, 13–15], challenges in meeting conduct and resistance to patient involvement [11, 13, 15], and discrepancies between the views of patients and physicians (the most commonly identified barrier in a knowledge synthesis) [13].

Potential barriers to successful external review and public comment include an insufficient understanding of the guideline development process, contradictory comments, and the resources and time required to collate and respond to comments [1]. Furthermore, while not described in the literature, the format of draft guidelines shared for external review and public comment is likely a major barrier to meaningful PPI. In this study, there was no evidence that developers prepare patient- and public-friendly guideline documents for draft review. With difficulty understanding medical terminology described as one of the most common barriers to PPI in guidelines [2, 11, 13–15], current public comment practices may be more tokenistic than meaningful engagement.

Finally, while many specialty organizations have plain-language educational pages on their websites targeting patients and families, only 20% of guideline developers create guideline-related plain-language products even some of the time. In an era where clinical practice guidelines are produced in ever-increasing numbers but implementation is variable and often poor [16, 17], this is another missed opportunity. Educational resources for patients and families and other tools for patient/family communication comprise one domain of a framework for guideline implementability to improve guideline use [18]. Research also shows that patient and family guideline summaries are a commonly accessed guideline implementation tool, second only to clinician summaries in a study of website accesses [19]. There is room for improvement even for those developers currently creating patient/public-targeted guideline materials, as limited evidence was found for patient and public engagement in the drafting/review process and this is an obvious opportunity for PPI [7, 11, 13].

This is the first study to systematically assess PPI approaches in guideline development based on review of published methodology rather than developer self-report. A potential limitation is the reliance on publicly accessible materials to assess guideline methodology. This approach was chosen a priori based on IOM transparency standards, but may have resulted in an under-estimate of PPI. Guideline manuals were available for only 44 (44%) of developers, so extractors relied on the methodology sections of recently published guidelines. This approach may have missed PPI if methodology sections were incomplete or if different guidelines employed different approaches. Finally, it is possible that guideline manuals and guidelines don’t reference every PPI strategy; for example, they might not describe a review of published patient preferences or patient engagement in guideline tool development even when these occur. While these limitations may result in an under-estimate of patient engagement, if guideline users are unaware of PPI (regardless of whether or not it occurred), there are implications for guideline trustworthiness.

## Conclusions

This study is an important step in understanding current PPI practices of U.S. guideline developers. Despite increasing discourse regarding PPI in guidelines, there remains a substantial gap between standards and practice. While frameworks [9, 20] and toolkits [4] for patient engagement exist, guideline developers must commit to prioritizing PPI as one element of trustworthy guideline development. The IOM tasked the Agency for Healthcare Research and Quality and the National Guideline Clearinghouse with providing “a clear indication of the extent to which clinical practice guidelines submitted adhere to the standards for trustworthiness” [1]. If guideline developers are unable or unwilling to meet standards for trustworthy guidelines on their own, the National Guideline Clearinghouse may need to make PPI a mandatory inclusion criterion to provide further impetus for improving practice.

## Additional files

Additional file 1: Description of data: Blank data extraction form. (XLSX 14 kb)

Additional file 2: Description of data: Final data set (consensus data set). (XLSX 97 kb)

## Abbreviations

AGREE II: Appraisal of Guidelines for Research and Evaluation II
CI: Confidence interval
G-I-N: Guidelines-International-Network
IOM: Institute of Medicine
NGC: National Guideline Clearinghouse
PPI: Patient and public involvement
RD: Risk difference
USPSTF: United States Preventive Services Task Force
